# Medical Service Utilization for Carpal Tunnel Syndrome in Korea (2010–2017): A Retrospective, Cross-Sectional Study Using a Nationally Representative Sample from the HIRA-National Patient Sample Database

**DOI:** 10.3390/healthcare14010109

**Published:** 2026-01-02

**Authors:** Ji Won Kim, Soo Jin Kim, Ye-Seul Lee, Yoon Jae Lee, In-Hyuk Ha, Ju Yeon Kim, Doori Kim

**Affiliations:** 1 Jaseng Hospital of Korean Medicine, 536 Gangnam-daero, Gangnam-gu, Seoul 06110, Republic of Korea; jiwon910425@gmail.com; 2 Jaseng Spine and Joint Research Institute, Jaseng Medical Foundation, 540 Gangnam-daero, Gangnam-gu, Seoul 06110, Republic of Korea

**Keywords:** carpal tunnel syndrome, healthcare utilization, national patient sample data, real-world data

## Abstract

**Background**: Carpal tunnel syndrome (CTS) is a common peripheral neuropathy with increasing prevalence and economic burden. This study aimed to analyze recent trends in CTS treatment patterns, healthcare utilization, and costs within the dualized healthcare system in Korea, using nationwide claim data. **Methods**: This cross-sectional study used data from the Korean Health Insurance Review and Assessment Service National Patient Sample (HIRA-NPS) between 2010 and 2017. Patients with a primary diagnosis of CTS (KCD-10: G56.0) were included. Descriptive analyses were performed to examine trends in patient characteristics, healthcare utilization, treatment patterns, and medical costs in Western and Korean medicine. **Results**: A total of 29,112 patients with CTS were analyzed. In Western medicine, diagnostic tests accounted for the highest expenditure, particularly X-ray, nerve conduction studies, and electromyography. Over time, X-ray utilization increased, while nerve conduction and electromyography tests decreased. The proportion of surgical treatment declined from 11.28% in 2010 to 8.55% in 2017, whereas Korean medicine use increased from 9.41% to 15.08%, mainly consisting of acupuncture and related procedures. **Conclusions**: Korea exhibited a lower CTS surgery rate than other countries, alongside a rising trend in Korean medicine utilization. These findings underscore the distinctive dual healthcare system in Korea and highlight the need for prospective studies to assess the long-term effectiveness of Korean medicine-based conservative treatments. Additionally, the results may inform national health policy decisions, including insurance coverage and resource allocation for CTS management.

## 1. Introduction

Carpal tunnel syndrome (CTS) is a peripheral nerve entrapment disorder caused by compression of the median nerve due to increased pressure within the carpal tunnel [1]. Symptoms include tingling and paresthesia on the outer surfaces of the thumb, 2nd, 3rd, and 4th fingers, which are sensory areas controlled by the median nerve, and may progress to weakened grip strength or impaired hand function [2]. Reported risk factors for CTS include female sex, age 40–49 years, high body mass index, and Raynaud’s syndrome [3].

Globally, approximately 4–5% of the population is affected, with the highest prevalence among individuals aged 40–60 years [4]. In a large United States survey, the prevalence of clinically diagnosed CTS among workers was 6.7% [5]. More than 500,000 carpal tunnel release surgeries are performed annually, generating healthcare costs exceeding $2 billion [6].

Because of Korea’s dual healthcare system, patients can choose between Western medicine (WM) and traditional Korean medicine (KM). WM treatment for CTS varies according to disease severity. For moderate cases, conservative approaches, such as splinting, local corticosteroid injection, prednisone, and physical therapy are considered [7]. However, these treatments are often associated with adverse effects [8,9,10], and a study comparing treatment preferences reported that patients were less inclined to choose surgery or corticosteroid injections than physicians [11]. Meanwhile, in a British NHS study, the proportion of patients with CTS undergoing surgery increased from 19.35% in 1993 to 27.41% in 2013, rising steadily until 2007 and declining thereafter [12]. These trends underscore the need to investigate the current usage patterns of medical service utilization for CTS.

Various KM therapies, including acupuncture, electroacupuncture, pharmacoacupuncture, warm acupuncture, laser acupuncture, acupotomy, Chuna therapy, herbal medicine, moxibustion, cupping, and physical therapy, are widely used as conservative treatments for CTS [13]. However, no comprehensive study has yet evaluated their current utilization status.

In Korea, approximately 0.29 per 1000 individuals underwent CTS surgery between 2005 and 2007 [14]. However, no studies have examined recent statistics or annual changes, and the use of KM in Korea is expected to differ from that in other countries. Therefore, research based on national health insurance claim data is needed to clarify current domestic medical utilization patterns.

With the prevalence of CTS and its associated economic burden continuing to rise, this study analyzed data from 2010 to 2017 to provide essential evidence for optimizing CTS management and informing future health insurance policies. Using claim data from the South Korean Health Insurance Review and Assessment Service (HIRA) National Patient Sample (NPS), the study examined patient characteristics, medical service utilization across WM and KM departments, medical costs, comorbidities, and temporal trends.

## 2. Materials and Methods

### 2.1. Data Source

HIRA-NPS data from 2010 to 2017 were used. Health insurance claims are generated when healthcare institutions submit reimbursement requests to the HIRA after providing healthcare services to a patient. HIRA releases de-identified annual sample datasets using stratified random sampling. Each dataset includes treatment and prescription records for all patients who used healthcare services in that year sampled systematically by 10-year age groups. Approximately 1.45 million patients are represented annually, corresponding to 3% of all patients in Korea [15].

### 2.2. Study Design and Population

The study included patients of all ages who visited WM or KM institutions with a primary diagnosis of CTS (KCD-10 code G56.0), based on the Korean Standard Classification of Diseases (KCD). The claim data contained information on services provided (e.g., treatments, procedures, examinations, and prescriptions), diagnoses, insurance payments, out-of-pocket costs, patient demographics, and institution type. The exclusion criteria comprised claims with billing form codes corresponding to relevant institutions (e.g., dental hospitals, midwifery centers, public health centers), missing or zero total cost, and cases with comorbidities potentially confounding CTS diagnosis, such as cervical disc disease with radiculopathy (M50.1), brachial plexus disorders (G54.0), nerve root and plexus compressions in intervertebral disc disorders (G55.1), and polyneuropathies or peripheral nervous system disorders (G60–G64).

### 2.3. Statistical Analysis

Descriptive statistics were used for analysis. Categorical variables were expressed as frequency (N) and percentage (%), while continuous variables were expressed as mean, standard deviation (SD), minimum (Min), and maximum (Max). Patients with CTS were classified by age (five groups in 10-year increments between 19 and 70 years), sex, payer type (National Health Insurance, Medicaid, and others), visit type (inpatient or outpatient), and institution type (tertiary/general hospital, hospital, clinic, KM hospital, or KM clinic). Frequencies within each category were analyzed.

The number of claims was calculated per hospital visit, and total expenditure was calculated as the sum of insurer-paid and out-of-pocket costs.

Annual trends in diagnostic testing were evaluated by counting the number of claims for nerve conduction studies, electromyography, manual muscle tests, hand function tests, and X-rays. The yearly proportion of patients receiving surgical or KM treatments against the total number of patients were also presented.

Medication use was analyzed using Anatomical Therapeutic Chemical (ATC) codes. Clinicians reviewed and classified medications by therapeutic purpose, including pain management (NSAIDs, opioid + non-opioid, opioid, and acetaminophen, musculoskeletal treatments (relaxants, other medications for musculoskeletal system), corticosteroids, and other indications unrelated to CTS. All statistical analyses were performed using SAS version 9.4 (SAS Institute Inc., Cary, NC, USA).

### 2.4. Cost Standardization

To ensure comparability of expenditures across years, all costs were standardized to 2020. All expenditures were first adjusted to 2020 values using the health sector Consumer Price Index, and converted from Korean won to US dollars using the annual average exchange rate for the corresponding year. We selected 2020 as a recent fixed baseline year to reflect the general price level after steady economic growth and inflation over the preceding decades, and to avoid incorporating pandemic-related effects in subsequent years. All monetary vales reported in this study are presented in 2020-adjusted USD.

## 3. Results

**Figure 1:**
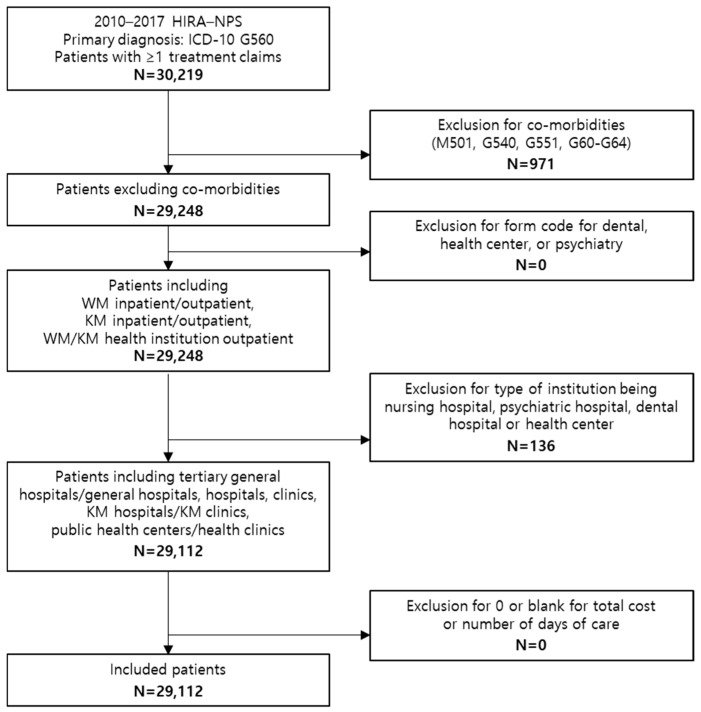
Flow chart of patient inclusion.

From 2010 to 2017, HIRA-NPS data initially included 30,219 patients with at least one record of receiving medical services for CTS (ICD-10 code G56.0) as the primary diagnosis. Patients with comorbidities associated with diagnosis codes M50.1, G54.0, G55.1, and G60–G64 (n = 971) were excluded. Additional exclusions included cases billed under dentistry or psychiatry (n = 0), claims from nursing or dental hospitals or with missing institution codes (n = 136), and those without total cost or length-of-care records (n = 0). Ultimately, 29,112 patients were included in the analysis (Figure 1).

Between 2010 and 2017, the number of CTS-related claims increased from 8811 to 13,516, while the number of patients with CTS increased from 2938 to 3946. The total number of patients increased significantly from 8822 in 2010 to 13,247 in 2012, stabilizing at around 13,000 thereafter. The number of patients using WM services mirrored the overall trends, while those utilizing KM services increased slightly from 2010 to 2014, and subsequently plateaued.

**Figure 2:**
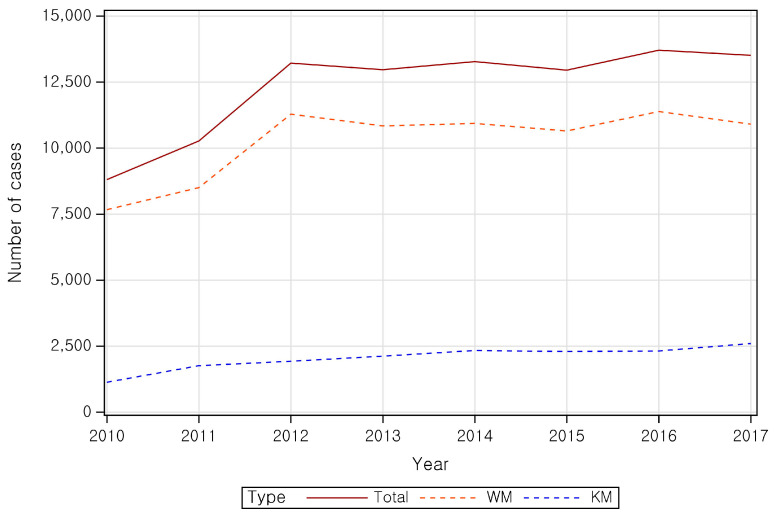
Number of CTS cases.

The total treatment cost of CTS increased from $648,024 in 2010 to $806,026 in 2012 and remained steady at around $800,000 through 2017 (Figure 2, Supplemental Table S1).

The total medical expense per patient for treatment of CTS fluctuated between $221 and $225 annually, with $221 in 2010 and $215 in 2017, with no significant trend.

**Figure 3:**
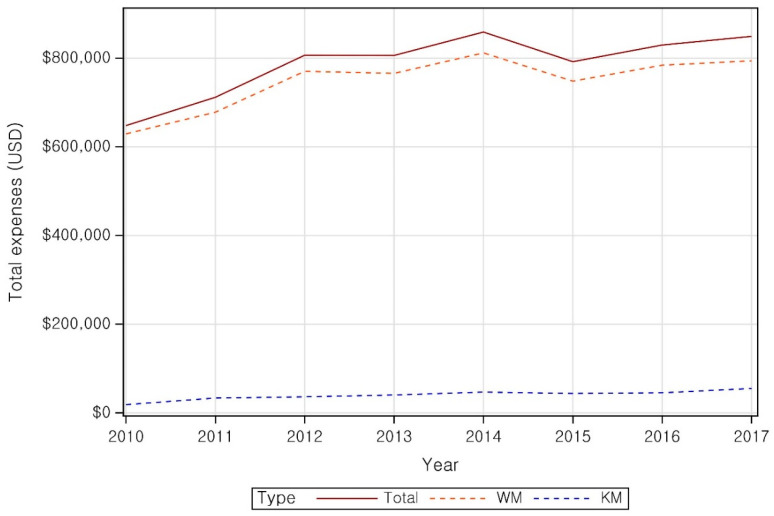
Total expense for CTS.

The medical expenses per patient for WM and KM institutions were $233 and $68, respectively, indicating approximately 3.4-times-greater expenses in WM institutions than in KM institutions. In 2017, the medical expense per patient for WM and KM institutions were $227 and $93, respectively, reflecting a gradual narrowing of the expenditure gap (Figure 3 and Supplemental Table S1).

Regarding demographics, female patients accounted for 78.29%, approximately 3.61 times more than male patients (21.71%). Most patients were covered by the NHI (95.28%), with only a small proportion of patients (4.69%) paid by medical aid.

When analyzed by age group, patients in their 50 s accounted for the highest proportion (38.62%), followed by those in their 40 s (21.32%) and 60 s (17.46%). Those aged < 19 years comprised the smallest group (0.66%).

Of the 29,058 patients with CTS between 2010 and 2017, 25,176 (86.7%) used WM services, 2975 (10.2%) used KM services, and 907 (3.1%) used both. Female predominance and the highest representation of patients in their 50 s were consistent across all groups. The combined WM + KM group showed a notably higher proportion of females (83.57%) and patients in their 50 s (44.10%) than those in the other groups (Table 1).

Outpatient visits comprised the majority (97.09%) across both WM and KM services. KM treatment was predominantly provided by primary care institutions (96.87%), whereas WM patients visited clinics (60.77%) and tertiary/general hospitals (39.23%), indicating a higher usage of advanced-level institutions in WM services (Table 2).

Analysis of claims by service type revealed that examination fees ($1,372,329.79), consultation fees ($1,049,983.12), and procedure and surgery fees ($1,000,951.22) accounted for the largest portions of total cost and expenses per patient.

For WM service users, the most frequent claims were consultation (81,338 cases), outpatient medication (41,731 cases), physical therapy (27,503 cases), and injections (24,446 cases), with average claims per patient of 3.12, 1.60, 1.05, and 0.94, respectively. Highest average medical expenses per patient were associated with examinations ($53), procedure and surgery ($38), consultations ($35), and admissions ($26) (Table 3).

Among KM-only patients, consultation claims were most frequent (16,524 cases), followed closely by injection (16,511 cases), and medication administration and prescription filling (1575 cases). The average claims per patient were 4.26 for consultations, 4.25 for injections, and 0.41 for medication services. Meanwhile, the average expenses per patient were highest for injections ($49.15), followed by consultations ($32.36) and medication administration and prescription filling fee (Table 3).

Diagnostic examinations most commonly used were X-rays, nerve conduction studies, electromyography, manual muscle tests, and hand function tests, in descending order of frequency. While these rankings remained consistent over time, usage proportions shifted: X-ray use increased from 26.5% in 2010 to 30.58% in 2017, nerve conduction studies declined from 25.99% in 2010 to 21.20% in 2017, and electromyography decreased slightly from 17.64% in 2010 to 14.80% in 2017 (Table 4).

**Figure 4:**
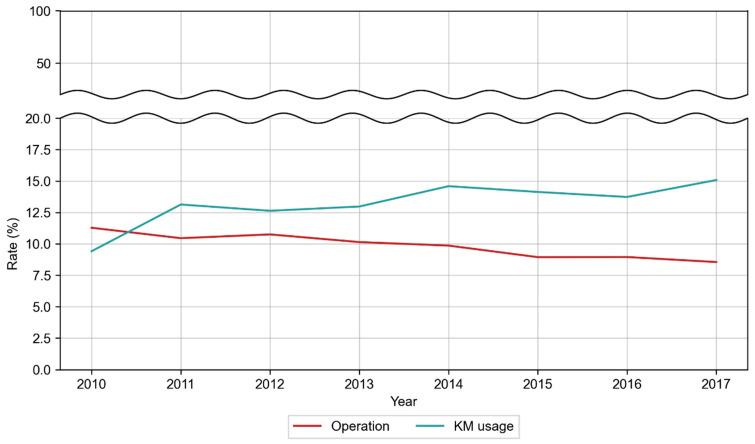
Rate of operation and KM usage for CTS.

Among those using WM services, the rate of operation declined from 11.28% in 2010 to 8.55% in 2017. Conversely, KM usage increased from 9.41% to 15.08% during the same period. After 2010, KM utilization consistently exceeded operation rates (Figure 4, Supplemental Table S2).

Table 5 summarizes annual outpatient medication prescriptions for patients with CTS. Alimentary tract and metabolic medications were most frequently prescribed, followed by non-steroidal anti-inflammatory drugs (NSAIDs), nervous system medications, other musculoskeletal drugs, and corticosteroids. Prescription trends showed overall increases for most medications, including NSAIDs (from 1749 cases in 2010 to 2321 cases in 2017), despite minor annual fluctuations. Opioid prescriptions declined from 452 cases in 2010 to 307 in 2017. Corticosteroid use rose gradually, moving from the fourth most prescribed medication between 2010 and 2013 to the third most prescribed from 2014 through 2017.

## 4. Discussion

This study utilized HIRA-NPS data from 2010 to 2017 to examine the distribution of patients with CTS according to demographic characteristics, treatment modalities, medical expenses, visit types, and annual trends, stratified by WM and KM institutions.

Among all patients with CTS who visited WM or KM institutions, women (78.29%) outnumbered men (21.71%) by 3.61 times. When compared by age group, those in their 50 s accounted for the highest proportion at 38.62%, followed by those in their 40 s (21.32%) and 60 s (17.46%). These findings are consistent with previous epidemiological studies on patients with CTS. For instance, an analysis of the UK General Practice Research Database in 2000 reported CTS incidences of 193 per 100,000 in women and 88 per 100,000 in men [2]. Other studies found annual incidence rates of 276 per 100,000, with higher rates among women (9.2%) than among men (6%) [16,17]. CTS is most prevalent among women aged 45–54 years, whereas men tend to be affected later, between 75 and 84 years [18]. Several explanations have been proposed for the female predominance in CTS. Dramatic changes in estrogen levels [19,20] and fat distribution [21] have been suggested as contributing factors, particularly in pregnant or postmenopausal women, and hormone replacement therapy has been reported to reduce CTS risk [22,23]. In addition, comorbidities that increase with age (e.g., diabetes, rheumatoid arthritis, and gout) [22] and exposure to vibration, repetitive wrist movement, cold, persistent external pressure or traction are associated with CTS development [20,24,25,26].

Across the WM, KM, and combined WM + KM groups, women predominated, and patients in their 50 s represented the largest age group. Notably, in the WM + KM group, women accounted for 83.57% and patients in their 50 s for 44.10%, higher than in the other two groups (Table 1). These results suggest that women may exhibit a more active attitude toward seeking diverse CTS treatments. A previous study on treatment predictors supports this observation, showing that women were more likely to receive additional injection therapy after undergoing carpal tunnel release (odds ratio 1.55) compared with men [27].

During the study period, outpatient care accounted for the majority (97.09%) of CTS-related visits, consistent with the non-restrictive mobility associated with the condition. The average medical expense per patient remained stable—from $221 in 2010 to $215 in 2017—without notable fluctuations ($210–$225). Examination ($1,372,329.79), consultation ($1,049,983.12), and procedure/surgery ($1,000,951.22) fees accounted for the largest portions of total expenditures.

Among WM services, examination fees also showed the highest average cost per patient ($53). The most frequently performed diagnostic examinations were X-ray, nerve conduction study, and electromyography, with consistent ranking throughout the study period. However, the proportion of patients undergoing each test changed: X-ray use increased from 26.50% to 30.58%, whereas nerve conduction study and electromyography decreased from 25.99% to 21.20% and from 17.64% to 14.80%, respectively. This pattern mirrors the declining use of preoperative electrodiagnostic studies (EDS) observed in the United States since 2014, following the American Academy of Orthopaedic Surgeons (AAOS) Clinical Practice Guideline (CPG) that de-emphasized routine EDS except in cases requiring surgical intervention. Considering the relatively low CTS operation rate and fewer severe cases in Korea, as well as the influence of the CPG, the observed decline in nerve conduction and electromyography testing appears consistent with these changes.

Regarding conservative management within WM, each patient with CTS received an average of 1.60 medication prescriptions, 1.05 physical therapy sessions, and 0.94 injections per year, reflecting standard treatment approaches for mild-to-moderate CTS.

The most frequently prescribed medications for patients with CTS at WM institutions were those for the alimentary tract and metabolism, followed by NSAIDs, which are presumed to prevent gastrointestinal side effects of NSAIDs. The number of NSAID prescriptions increased from 1749 in 2010 to 2321 in 2017. From 2010 to 2013, “other medications for the musculoskeletal system” ranked third, but with the increase in corticosteroid prescriptions, corticosteroids surpassed them from 2014 to 2017, ranking next to NSAIDs.

Pharmacotherapy for CTS commonly includes NSAIDs, corticosteroids, diuretics, and pyridoxine [28]. Some studies have reported that oral corticosteroids are less effective than corticosteroid injections [21], while others found oral steroids to be as effective as intracarpal steroids in improving nerve conduction parameters [29]. However, the latest AAOS clinical practice guideline for CTS management [30] downgraded the strength of recommendation for long-term oral steroid use to ‘limited’ [30]. A survey of hand surgeons in the US reported that over 80% of the surgeons do not consider oral steroids beneficial for CTS treatment [31]. Further studies are needed to assess current patterns and trends in long-term oral corticosteroid use for CTS.

The operation rate among patients with CTS receiving WM services decreased from 11.28% in 2010 to 8.55% in 2017. Before 2010, Korea’s CTS surgery rate was already lower than in other countries. A nationwide cohort study using HIRA data from 2005 to 2007 reported an operation rate of 0.29 per 1000 person-years, compared with 0.45–1.48 per 1000 person-years in Western countries, such as Sweden, Finland, and the United Kingdom during the same period [14]. These findings suggest that, even before 2010, distinctive treatment patterns in Korea had been shaped by structural and cultural factors.

The low surgical rate in Korea likely reflects multiple factors. The coexistence of WM and KM systems provides patients with diverse non-surgical options for mild to moderate CTS, including splinting, steroid injections, pharmacotherapy, and physical therapy in WM, as well as acupuncture, pharmacopuncture, Chuna therapy, moxibustion, and herbal medicine in KM. The accessibility of these non-invasive options naturally contributes to low surgical intervention rates.

Social and cultural perceptions also influence this trend. Although carpal tunnel release is safe, conservative treatment is often preferred when symptoms are mild or do not significantly interfere with daily life. Concerns about postoperative recovery and potential loss of income further discourage surgery. Conversely, KM treatment offer short recovery periods and minimal disruption to daily life, promoting treatment continuity.

Operation rates also varied by sex and region. Young et al. [14] reported that women underwent CTS surgery at roughly twice the rate of men, reflecting both higher prevalence and hormonal or occupational influences. Regionally, surgical rates were lower in rural areas than in metropolitan areas, likely due to limited access to specialized surgical care and higher KM utilization. These findings indicate that Korea’s low surgical rate stems not from differences in medical advancement, but from complex interactions among healthcare structure, culture, economics, and treatment philosophy.

Compared with Western countries, this difference is striking. For example, a study conducted by the National Health Service in the UK with an extensive collection of primary care data on patients with CTS from 1993 to 2013 reported that 27.41% of patients with CTS underwent surgery in 2013 [12]. The rate of operation steadily increased from 1993 to 2007 before stabilizing, contrasting sharply with Korea’s rate of 0.29 per 1000 person-years. Even accounting for population and diagnostic differences, Korea’s reliance on conservative management rather than surgical treatment remains evident.

CTS treatment in Korea exhibited a distinctive pattern shaped by high accessibility to non-surgical options, patient perceptions of surgery, and the structural characteristics of a dual healthcare system. This context provides an important foundation for interpreting the trends in healthcare utilization for CTS between 2010 and 2017.

Previous studies comparing surgical and conservative treatments for CTS found that surgery offered superior short-term outcomes in functional status and nerve conduction at 6 months, but no significant differences were observed at 3 or 12 months [32]. However, surgery is associated with the risk of nerve injury, including the median nerve [33], and endoscopic carpal tunnel release has been associated with a higher, albeit mostly temporary, risk of neurapraxia compared with open carpal tunnel release [34]. Another study reported that surgery was more effective than local steroid injection in the short term, but 58% of patients in the injection group required no further treatment during long-term follow-up [35]. Therefore, surgical decisions should be made through a comprehensive assessment of complication risks and patient preferences.

Given Korea’s extensive health insurance coverage and access to medical services, the low operation rate observed in this study likely reflects the wide availability of conservative treatment modalities. Since KM treatments are covered by national health insurance and their use among patients with CTS increased steadily during the study period, KM likely contributed to the low operation rate to some extent.

In 2010, the medical expense per patient for CTS treatment was approximately 3.4 times higher in WM institutions than in KM institutions, decreasing to 2.4 times higher by 2017. The expense gap between WM and KM institutions showed a declining trend. KM usage rose from 9.41% in 2010 to 15.08% in 2017, surpassing the operation rate after the first study year. Among KM users, the average per-patient expense was highest for injection fees ($49.15), followed by consultation fees ($32.36), and medication administration and prescription filling fees ($0.81). On average, KM patients received 4.25 injections and 0.41 medication administration and prescription filling compared to 1.60 outpatient medication costs, 1.05 physical therapy sessions, and 0.94 injections among WM patients. These data indicate that KM patients undergo more consecutive conservative treatments per case.

Injection, which includes acupuncture, was the most frequently prescribed KM service for CTS, consistent with prior reports that KM institutions typically provide acupuncture, moxibustion, cupping, and herbal medicine [13]. A national survey of Korean medicine doctors that acupuncture had the highest patient satisfaction rate (90.3%) and was perceived as the most effective treatment (90.8%) for CTS [13], demonstrating strong patient trust in its effectiveness.

Clinical studies also support the efficacy of KM treatments. One study found that acupuncture provided greater pain relief than ibuprofen, with fewer side effects [9], and a randomized controlled trial reported that acupuncture reduced nocturnal awakenings from pain and distal motor latency compared to prednisolone [10]. Recent studies have confirmed that acupuncture and electroacupuncture therapy for CTS improve pain, nerve conduction, and physical function. Acupuncture and electroacupuncture have been reported to inhibit pain signal transmission and promote nerve regeneration by modulating neurotransmitters and inflammatory mediators in the peripheral and central nervous systems.

Electroacupuncture contributes to pain relief and functional improvement by promoting the release of endogenous opioid peptides and activating descending pain-inhibitory pathways. Han [36] reported that low-frequency (2–10 Hz) stimulation releases β-endorphin, enkephalin, and dynorphin, which bind to μ/δ/κ receptors in the spinal cord and central nervous system to inhibit pain signaling pathway. In this process, selective activation of Aβ/Aδ fibers is involved in gate control, reducing the transmission of pain signals in the dorsal horn. Zhao [30] further demonstrated that electroacupuncture activates inhibitory interneurons and NMDA/substance P regulation in the dorsal horn, strengthens downstream inhibitory pathway involving the midbrain periaqueductal gray (PAG) and the nucleus raphe magnus/locus coeruleus (NRM/LC) in the brain stem, and the interaction between the serotonin (5-HT)-norepinephrine (NE) system and the opioid system [37]. This downstream pathway, linked to the medial pain modulatory network, is functionally activated by electroacupuncture stimulation, resulting in the inhibition of pain conduction and remodulation of central nervous system pain perception at the spinal cord level. In the context of CTS, this mechanism alleviates peripheral compression-induced hyperalgesia, leading to reduced nighttime pain and numbness/tingling sensations and improved functional status (from the Boston Carpal Tunnel Questionnaire). In practice, low-frequency mixed waveforms are advantageous for inducing the release of endogenous opioid peptides when applied to wrist acupoints, LI4, and PC6, and the synergistic effects may be expected when combined with splints and work modification. Collectively, these findings support electroacupuncture as an adjunctive treatment for CTS. Nonetheless, further randomized controlled trials are warranted to confirm its long-term efficacy and optimize treatment parameters across patient subgroups.

Zhang et al. reported that electroacupuncture alleviates neuropathic pain by increasing the release of endorphins, serotonin, and norepinephrine in the dorsal horn and central nervous system while suppressing the expression of pain mediators, such as TNF-α, IL-1β, and IL-6 [38]. Activation of this endogenous pain-modulating system helps reduce pain perception and alleviate median nerve hyperexcitation in compressive neuropathy associated with CTS. Furthermore, a study demonstrated that grip strength in the affected hand significantly improved after acupuncture [32], suggesting that acupuncture may enhance median nerve motor function.

Using HIRA NPS data, we investigated the current status of CTS diagnosis and treatment in Korea. However, this study has several limitations. First, analyses of non-benefit items, such as pharmacopuncture, herbal medicine, and Chuna manual therapy were not included. Notably, Chuna therapy has been covered by national health insurance only since 2018, and a prior study reported that its clinical outcomes were comparable to surgery at 1- and 4-year follow-ups [39], underscoring the need for further studies on its utilization. Second, as the HIRA-NPS consists of annual sample data, long-term follow-up analyses were not feasible, limiting assessment of outcomes among surgical and non-surgical patients. For example, revision surgery or recurrent CTS after carpal tunnel release could be identified only if it occurred within the same calendar year, and revisions beyond the index year were not reliably captured; thus, we could not evaluate surgical failure or characterize comorbidities among revision cases. Given the continued use of conservative treatments even after surgery for CTS [40], further research is needed to examine long-term outcomes and patient needs. Finally, the absence of disease severity data precluded analyses of healthcare utilization by disease severity, limiting interpretation of treatment outcomes by clinical status.

The operation rate for CTS in Korea was markedly lower than that in other countries, while KM treatment utilization showed a continuous increase. Despite its limitations, this study provides valuable insights into how CTS treatment is implemented within Korea’s national health insurance system, which uniquely integrates WM and KM. Future prospective studies are needed to establish robust evidence on the efficacy and operation rates of conservative treatments, including KM treatments, such as acupuncture, pharmacopuncture, and Chuna therapy. Research exploring decision-making processes and long-term treatment outcomes by disease severity will also help inform personalized CTS management strategies.

## 5. Conclusions

This study analyzed eight years of HIRA-NPS claim data to evaluate healthcare utilization among patients with CTS in Korea, comparing treatment types, visit frequency, and medical costs between WM and KM institutions. As no previous studies have examined CTS healthcare utilization at the national level, these findings provide a valuable reference for patient care and treatment planning. Moreover, they may serve as an evidence base for national health policy decisions, including insurance coverage and resource allocation for CTS management.

## Figures and Tables

**Table 1:** Basic characteristics of patients.

Category		Patient							
		Total (2010–2017)		WM (2010–2017)		KM (2010–2017)		WM + KM (2010–2017)	
		Total N	Percent	Total N	Percent	Total N	Percent	Total N	Percent
		29,058	100.00	25,176	100.00	2975	100.00	907	100.00
Sex	Male	6310	21.72	5462	21.70	699	23.50	149	16.43
	Female	22,748	78.28	19,714	78.30	2276	76.50	758	83.57
Age	≤19	193	0.66	168	0.67	25	0.84	0	0.00
	20–29	1074	3.7	903	3.59	154	5.18	17	1.87
	30–39	2546	8.76	2141	8.50	339	11.39	66	7.28
	40–49	6194	21.32	5374	21.35	616	20.71	204	22.49
	50–59	11,222	38.62	9832	39.05	990	33.28	400	44.10
	60–69	5073	17.46	4380	17.40	542	18.22	151	16.65
	≥70	2756	9.48	2378	9.45	309	10.39	69	7.61
Payer type	NHI	27,688	95.29	23,926	95.03	2884	96.94	878	96.80
	Medicaid	1363	4.69	1243	4.94	91	3.06	29	3.20
	Others *	7	0.02	7	0.03	0	0.00	0	0.00

* Veterans’ health insurance.

**Table 2:** Basic characteristics of medical usage.

Category		Claims					
		Total (2010–2017)		WM (2010–2017)		KM (2010–2017)	
		Total N	Percent	Total N	Percent	Total N	Percent
		98,737	100.00	82,200	100.00	16,537	100.00
Type of visit	Outpatient	95,858	97.08	79,329	96.51	16,529	99.95
	Inpatient	2879	2.92	2871	3.49	8	0.05
Medical institution	Tertiary hospital/ general hospital/hospital	32,246	32.66	32,212	39.19	34	0.21
	Clinic	49,941	50.58	49,941	60.76	0	0.00
	KM hospital	516	0.52	31	0.04	485	2.93
	KM clinic	16,015	16.22	0	0.00	16,015	96.84
	Public health center/health clinic	19	0.02	16	0.02	3	0.02

**Table 3:** Number of bills and medical costs per service category.

	Total						WM						KM					
Category	Case	%	Case per Patient	Cost	%	Cost per Patient	Case	%	Case per Patient	Cost	%	Cost per Patient	Case	%	Case per Patient	Cost	%	Cost per Patient
Examination fee	12,215	4.49	0.42	$1,372,330	21.55	$47	12,215	5.15	0.47	$1,372,330	22.70	$53	-	-	-	-	-	-
Anesthesia fee	15,776	5.80	0.54	$571,934	8.98	$20	15,688	6.61	0.60	$571,559	9.45	$22	88	0.25	0.02	$375	0.12	$0
Non-benefit services/others	1221	0.45	0.04	$29,872	0.47	$1	1221	0.51	0.05	$29,872	0.49	$1	-	-	-	-	-	-
Imaging diagnosis and radiology	11,538	4.24	0.40	$246,482	3.87	$8	11,538	4.86	0.44	$246,482	4.08	$9	-	-	-	-	-	-
Outpatient medication costs	41,731	15.34	1.44	$541,409	8.50	$19	41,731	17.59	1.60	$541,409	8.96	$21	-	-	-	-	-	-
Physical therapy fee	27,503	10.11	0.95	$182,925	2.87	$6	27,503	11.59	1.05	$182,925	3.03	$7	-	-	-	-	-	-
Admission fee	2859	1.05	0.10	$688,363	10.81	$24	2854	1.20	0.11	$685,713	11.34	$26	5	0.01	0.00	$2650	0.82	$1
Fee for psychotherapy	9	0.00	0.00	$212	0.00	$0	9	0.00	0.00	$212	0.00	$0	-	-	-	-	-	-
Injection fee	40,957	15.06	1.41	$587,408	9.22	$20	24,446	10.30	0.94	$396,612	6.56	$15	16,511	47.58	4.25	$190,796	59.14	$49
Consultation fee	97,862	35.98	3.37	$1,049,983	16.49	$36	81,338	34.28	3.12	$924,363	15.29	$35	16,524	47.62	4.26	$125,620	38.94	$32
Procedure and surgery	11,446	4.21	0.39	$1,000,951	15.72	$34	11,446	4.82	0.44	$1,000,951	16.56	$38	-	-	-	-	-	-
Medication administration and prescription filling fee	8862	3.26	0.3	$96,523	1.52	$3	7287	3.07	0.28	$93,363	1.54	$4	1575	4.54	0.41	$3160	0.98	$1
All	271,979	100	9.36	$6,368,393	100	$219	237,276	100.00	9.10	$6,045,792	100.00	$232	34,703	100.00	8.94	$322,601	100.00	$83

**Table 4:** Number of patients with diagnostic examinations for CTS.

Items	Number of Patients N (%)							
	2010	2011	2012	2013	2014	2015	2016	2017
Nerve condition study	765 (25.99%)	822 (25.34%)	947 (25.50%)	905 (24.01%)	856 (22.75%)	803 (21.48%)	825 (20.68%)	838 (21.20%)
Electromyography	519 (17.64%)	570 (17.57%)	704 (18.96%)	651 (17.27%)	618 (16.43%)	585 (15.65%)	588 (14.74%)	585 (14.80%)
Manual muscle test	24 (0.82%)	22 (0.68%)	24 (0.65%)	28 (0.74%)	13 (0.35%)	17 (0.45%)	13 (0.33%)	26 (0.66%)
Hand function test	8 (0.27%)	13 (0.40%)	9 (0.24%)	9 (0.24%)	12 (0.32%)	7 (0.19%)	15 (0.38%)	4 (0.10%)
X-ray	780 (26.50%)	879 (27.10%)	1094 (29.46%)	1054 (27.96%)	1050 (27.91%)	1052 (28.14%)	1186 (29.72%)	1209 (30.58%)
Ultrasound	0 (0%)	0 (0%)	0 (0%)	0 (0%)	0 (0%)	0 (0%)	2 (0.05%)	1 (0.03%)
Arthrography	0 (0%)	0 (0%)	1 (0.03%)	2 (0.05%)	0 (0%)	0 (0%)	0 (0%)	0 (0%)
CT	1 (0.03%)	3 (0.09%)	2 (0.05%)	8 (0.21%)	6 (0.16%)	5 (0.13%)	14 (0.35%)	5 (0.13%)
Bone scan	0 (0%)	1 (0.03%)	3 (0.08%)	0 (0%)	0 (0%)	0 (0%)	1 (0.03%)	0 (0%)
SPECT	0 (0%)	1 (0.03%)	1 (0.03%)	0 (0%)	0 (0%)	0 (0%)	1 (0.03%)	0 (0%)
MRI	0 (0%)	0 (0%)	0 (0%)	0 (0%)	1 (0.03%)	0 (0%)	1 (0.03%)	0 (0%)
Raynaud’s scan	2 (0.07%)	0 (0%)	0 (0%)	0 (0%)	2 (0.05%)	2 (0.05%)	0 (0%)	0 (0%)

**Table 5:** Frequently administered medications for CTS.

		Total	2010	2011	2012	2013	2014	2015	2016	2017
1	Alimentary tract and metabolism	17,277	1777	1897	2222	2260	2222	2183	2364	2352
2	Non-steroidal anti-inflammatory drug	16,960	1749	1855	2180	2210	2156	2164	2325	2321
3	Nervous system	12,104	1204	1300	1506	1607	1527	1585	1680	1695
4	Other medications for musculoskeletal system *	8401	854	910	1060	1073	1071	1033	1234	1166
5	Corticosteroids	8278	737	858	976	1060	1092	1125	1218	1212
6	Blood and blood forming organs	7627	869	903	932	930	940	958	1058	1037
7	Muscle relaxants	6322	730	754	907	884	765	763	761	758
8	Opioids in combination with non-opioid analgesics	3673	210	386	497	507	498	506	541	528
9	Anti-infectives for systemic use	3343	396	384	463	443	429	406	425	397
10	Opioids	2787	452	313	358	362	347	306	342	307
11	Others	1989	231	234	264	257	257	258	243	245
12	Acetaminophen	1583	182	169	202	196	208	212	201	213
13	Antineoplastic and immunomodulating agents	1191	147	139	141	161	175	143	133	152
14	Dermacologicals	233	30	34	23	29	30	26	27	34
15	Drugs for diabetes	180	18	16	27	29	24	24	22	20
16	Ophthalmologicals	75	6	2	10	19	9	12	10	7
17	Genito urinary system and sex hormones	73	8	7	6	11	8	6	13	14
18	Systemic hormonal preparations, excluding sex hormones and insulins	30	4	4	3	5	9	2	2	1

* Musculoskeletal system-enzymes, other drugs for disorders of the musculoskeletal system, Drugs for treatment of bone diseases.

## Data Availability

Data is unavailable due to ethical restrictions.

## Supplementary Materials

The following supporting information can be downloaded at: https://www.mdpi.com/article/10.3390/healthcare14010109/s1. Table S1: Number of CTS cases and patients by medical usage group and expense per patient; Table S2: Rate of operation and KM usage for CTS.
