# Supplementary material for: Medical Service Utilization for Carpal Tunnel Syndrome in Korea (2010–2017): A Retrospective, Cross-Sectional Study Using a Nationally Representative Sample from the HIRA-National Patient Sample Database
Source: Healthcare (Basel). 2026 Jan 2;14(1):109. doi: 10.3390/healthcare14010109 (PMC12785344; doi:10.3390/healthcare14010109)
Supplement: Supplementary file 1 [file healthcare-14-00109-s001.zip › healthcare-4039742-supplementary.pdf]

Supplemental Table S1. Number of CTS cases and patients by medical usage group and expense per patient

Year	Group	Total case	Total patient	Total expense	Total expense per patient (mean ± standard deviation)
2010	Total	8811	2938	$648,024	$221 ± 406
2010	WM	7672	2705	$629,322	$233 ± 419
2010	KM	1139	277	$18,702	$68 ± 87
2011	Total	10274	3238	$711,863	$220 ± 387
2011	WM	8508	2912	$678,217	$233 ± 402
2011	KM	1766	426	$33,647	$79 ± 115
2012	Total	13221	3706	$806,800	$218 ± 386
2012	WM	11287	3346	$770,447	$230 ± 401
2012	KM	1934	469	$36,352	$78 ± 112
2013	Total	12971	3759	$806,203	$214 ± 382
2013	WM	10844	3382	$765,757	$226 ± 396
2013	KM	2127	489	$40,446	$83 ± 113
2014	Total	13279	3754	$858,983	$229 ± 413
2014	WM	10938	3328	$811,960	$244 ± 432
2014	KM	2341	549	$47,023	$86 ± 111
2015	Total	12955	3733	$792,103	$212 ± 410
2015	WM	10653	3327	$748,230	$225 ± 427
2015	KM	2302	528	$43,874	$83 ± 124
2016	Total	13710	3984	$829,693	$208 ± 367
2016	WM	11387	3578	$784,231	$219 ± 381
2016	KM	2323	548	$45,462	$83 ± 95
2017	Total	13516	3946	$849,217	$215 ± 387
2017	WM	10911	3505	$794,024	$227 ± 401
2017	KM	2605	596	$55,193	$93 ± 138

Supplemental Table S2. Rate of operation and KM usage for CTS

	2010	2011	2012	2013	2014	2015	2016	2017
Total patient	2,943	3,244	3,713	3,769	3,762	3,738	3,990	3,953
Number of operated patients	332	339	399	382	371	334	357	338
Rate of operation (%)	11.28	10.45	10.75	10.14	9.86	8.94	8.95	8.55
Number of patients who used KM	277	426	469	489	549	528	548	596
Rate of KM usage (%)	9.41	13.13	12.63	12.97	14.59	14.13	13.73	15.08
